# Level of radiographic damage and radiographic progression are determinants of physical function: a longitudinal analysis of the TEMPO trial

**DOI:** 10.1136/ard.2007.081331

**Published:** 2008-01-18

**Authors:** D van der Heijde, R Landewé, R van Vollenhoven, S Fatenejad, L Klareskog

**Affiliations:** 1 Leiden University Medical Centre, Leiden, The Netherlands; 2 University Hospital, Maastricht, The Netherlands; 3 Karolinska University Hospital, Stockholm, Sweden; 4 Wyeth Research, Collegeville, Pennsylvania, USA

## Abstract

**Background:**

Many studies have examined the relationship between long-term radiographic damage and physical function. However, it is not known if short-term radiographic progression is also associated with physical function.

**Aim:**

To investigate the longitudinal relationship between physical function and both the level of radiographic damage and the radiographic progression rate in patients with early or advanced active rheumatoid arthritis.

**Methods:**

The database for the 2-year Trial of Etanercept and Methotrexate with Radiographic Patient Outcomes (TEMPO) was used for this study. Physical function was measured by the Health Assessment Questionnaire (HAQ) score at baseline, 6 months and 1 and 2 years. Radiographs of the hands and feet, taken at the same time points, were scored by the van der Heijde-modified Total Sharp Score (TSS). The HAQ score was modelled using generalised mixed linear modelling by TSS or progression in TSS (interval 0–1 year and 1–2 years) adjusted for age, sex, treatment and disease activity.

**Results:**

After adjustment for age, sex and disease activity, both TSS and the change in TSS (progression rate) were significant determinants of the HAQ score. When radiographic progression was divided into four categories (negative, zero, minor and greater progression), results showed that HAQ scores tended to be higher with a higher rate of progression. Patients with negative progression scores had lower HAQ scores than patients with positive progression scores.

**Conclusions:**

Patients with greater radiographic damage, and those with recent radiographic progression, have a higher degree of disability.

Rheumatoid arthritis (RA) affects almost 1% of the adult population.^1^ Affected subjects experience considerable morbidity, including a rapid loss of function,^2^ ^3^ joint destruction, permanent deformity and reduced life expectancy.^4^ ^5^ Based on published guidelines,^6^ ^7^ treatment of RA should focus on preserving function, preventing or controlling joint damage and achieving remission of disease activity.

Progressive radiographic damage and deterioration in physical function occur in patients with RA who do not receive timely effective treatment. Several long-term prospective studies have looked at the relationship between radiographic damage and physical function in patients with RA.^8^^–^10 A prospective 6-year follow-up study by Kuper *et al*^10^ showed that large joint damage occurs early in the disease, with at least 20% of patients having damage in at least one joint within 1 year of disease onset and 50% of patients having some damage within 6 years of disease onset. Radiographic changes were significantly related to damage in the hands and feet, the physical disability index and cumulative disease activity.^10^ In a review of the links between joint damage and disability in patients with RA, Scott *et al* reported that joint damage progresses continuously over the first 20 years of RA and that this damage accounts for 25% of the disability seen in patients.^9^ Radiographic damage is thus clearly a major determinant of long-term physical function in patients with established RA.

Although studies have shown that a relationship between physical function and radiographic damage exists, they have mostly examined this association looking at radiographic damage over the long term. It is not known whether progression of radiographic damage over short periods is associated with immediate impairment in physical function. This question is particularly relevant today because of the availability of powerful treatments that can prevent radiographic progression if started early enough. For example, in clinical trials, etanercept, either alone or in combination with methotrexate, has been shown to reduce radiographic progression in patients with recent and long-term RA.^11^^–^13

Our study used data from the Trial of Etanercept and Methotrexate with Radiographic Patient Outcomes (TEMPO),^11^ ^14^ to investigate the longitudinal relationship between physical function and both level of radiographic damage and radiographic progression over a 2-year period, in a cohort of patients with early or advanced active RA.

## METHODS

TEMPO was a multicentre, double-blind study that compared the effect of etanercept plus methotrexate versus etanercept alone versus methotrexate alone in 686 patients with active RA of 6 months to 20 years’ duration.^11^ ^14^ Details of this trial are published elsewhere.^11^ ^14^^–^16

All patients were randomly assigned to one of three treatment groups: etanercept (25 mg twice-weekly subcutaneous doses), methotrexate (7.5–20 mg weekly oral doses) or combination therapy with etanercept and methotrexate.

Physical function was measured by the Health Assessment Questionnaire (HAQ) at baseline and at protocol-specified intervals throughout the study; scores at baseline and 1 and 2 years were used in the analysis. The HAQ is scored on a scale of 0–3, with higher scores indicating increased disability.

Radiographs of the hands, wrists and forefeet were taken at baseline, 6 months, 1 year and 2 years. Digitised images were scored by two readers, using the van der Heijde-modified Sharp method.^17^ Radiographic progression was determined for each interval by subtracting status scores of two time points. Radiographic progression was divided into four categories (negative, <0; zero, 0–1; minor, 1–5; and greater progression, >5) for comparisons with HAQ scores. The radiographic population consisted of patients who had an acceptable baseline and at least one acceptable post-baseline film.

### Statistical analysis

To control for within-patient correlation, generalised mixed linear modelling was used to model the dependent variable HAQ score by the absolute Sharp score (damage score) or the interval change in Sharp score, with age, sex, disease duration, treatment, Disease Activity Score (DAS) and C-reactive protein (CRP) levels as covariates. If interval change in Sharp score was entered as an independent variable, the HAQ score at the end of the interval was chosen as the dependent variable in the model and the HAQ score at baseline was omitted. A random intercept and a compound symmetry covariance structure seemed to best fit the data. Results are expressed as estimated marginal means. In separate analyses, the interaction of disease duration and (change in) Sharp score with respect to HAQ score was tested.

## RESULTS

Of the 686 randomly assigned patients, 622 (91%) had a baseline and at least one follow-up film and were included in the radiographic analysis. No statistically significant differences were found among the three treatment groups in baseline demographic and disease characteristics. Patients included in the analysis were predominantly women (76.5%), with mean disease duration of 6.35 years, mean HAQ score of 1.72 and mean total Sharp score (TSS) of 33.0 at baseline.

In the linear mixed model, which was applied here in order to aggregate data of baseline, year 1 and year 2 data under simultaneous adjustment for within-patient correlation, the TSS was significantly positively associated with the HAQ score independently of the DAS (table 1). A number of additional variables were independently associated with the HAQ score, including age (positive correlation), sex (women had higher HAQ scores) and the DAS (positive correlation).

**Table 1:** Sharp score is positively associated with Health Assessment Questionnaire (HAQ) score independently of Disease Activity Score (DAS): generalised mixed linear modelling*

Parameter	Regression coefficient	p Value	95% CI of the regression coefficient	
			Lower bound	Upper bound
Intercept	−0.125	0.26	−0.342	0.092
Sex (F)	−0.243	<0.001	−0.333	−0.152
Age (years)	0.0103	<0.001	0.0073	0.0133
Disease duration (years)	0.0059	0.174	−0.0026	0.0143
CRP (mg/dl)	0.0029	<0.001	0.0014	0.0044
Treatment	0.0104	0.67	−0.0369	0.0577
DAS	0.255	<0.001	0.240	0.2688
Sharp score (Sharp units)	0.0019	<0.001	0.0011	0.0027

*Dependent variable: HAQ.

CRP, C-reactive protein.

**Figure 1:**
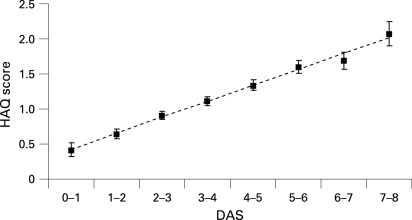
Marginal means for the Health Assessment Questionnaire (HAQ) score, adjusted for age, sex, disease duration, treatment, Sharp score and time as a function of disease activity score (DAS) in categories of one unit (error bars reflect standard error).

The HAQ score is primarily determined by disease activity. In the context of a clinical trial, it is expected that the disease activity would show important variation that would easily obscure a contributory and independent association with radiographic damage or progression. We first investigated the relationship between the DAS and the HAQ score. After adjustment for age and sex, the relationship between DAS and the HAQ score was almost linear (fig 1). This linear relationship justifies why in every subsequent analysis to determine the contribution of radiographic damage to explaining physical function we have adjusted for the known relationship between the DAS and the HAQ score.

**Figure 2:**
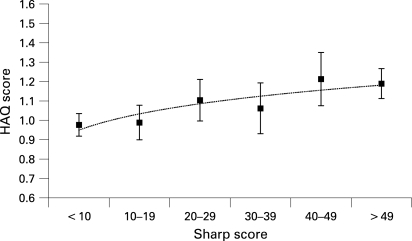
Marginal means for the Health Assessment Questionnaire (HAQ) scores adjusted for age, sex, disease duration, treatment, Disease Activity Score (DAS), Sharp score and time as a function of increasing Sharp scores (in categories of 10 units; error bars reflect standard error).

To visualise the adjusted relationship between the HAQ score and the Sharp score, Sharp scores were divided into six categories of 10 Sharp units each and estimated marginal means were calculated. Figure 2 shows estimated marginal means of the fitted model (mean HAQ scores adjusted for age, sex, DAS, treatment and time) for each category of 10 Sharp units and clearly points to an increasing trend. A separate analysis, in which the Sharp score was replaced by the change in Sharp score, also indicated that the change in Sharp score (interval progression; Sharp units per year) was significantly and independently associated with the HAQ score (p = 0.001; table 2). This implies that patients who show joint damage progression have worse physical function, independent of disease activity and the type of treatment they use, although the effects were small.

**Table 2:** Health Assessment Questionnaire (HAQ) score is positively associated with radiographic progression rate independently of disease activity (Disease Activity Score (DAS) and C-reactive protein (CRP)): generalised mixed linear modelling*

Parameter	Regression coefficient	p Value	95% CI of the regression coefficient	
			Lower bound	Upper bound
Intercept	−0.0142	0.922	−0.3007	0.2723
Sex (F)	−0.3118	<0.001	−0.4307	−0.1928
Age (years)	0.0120	<0.001	0.0080	0.0159
Disease duration (years)	0.0113	0.057	−0.0015	0.0241
CRP (mg/dl)	0.0024	0.085	0.0003	0.0051
Treatment	0.0026	0.934	−0.0997	0.0649
DAS	0.2313	<0.001	0.1969	0.2658
Change in Sharp score (Sharp units/year)	0.0088640	0.001	0.0035	0.0143

*Dependent variable: HAQ

**Figure 3:**
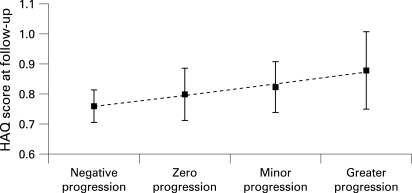
Marginal means for the Health Assessment Questionnaire (HAQ) score, adjusted for age, sex, disease duration, treatment, disease activity score (DAS), Sharp score and time, as a function of change in Sharp score (four categories of progression; error bars reflect standard error).

Figure 3 shows the effect of changes in radiographic damage on physical function, with progression of radiographic damage stratified into four categories (negative, zero, minor and greater progression). HAQ scores tended to increase with increasing radiographic progression, although the differences were small and probably not clinically meaningful. Figure 3 also suggests that patients with negative progression scores have lower HAQ scores than patients with positive progression scores, but this effect was not statistically significant.

In an attempt to determine whether the documented association between the Sharp score and the HAQ score, as well as the association between the change in Sharp score and HAQ score, was dependent on disease duration, we tested the following interactions: Sharp score and disease duration and change in Sharp score and disease duration, with disease duration dichotomised at a cut-off level of 3 years, with respect to explaining variation in HAQ score. Both interactions were not statistically significant (results not shown)

## DISCUSSION

This analysis provides further evidence for the longitudinal relationship between radiographic damage and physical function. As shown, progression of radiographic damage over short periods of time is associated with worse physical function, which is independent of the effects of inflammatory disease activity (DAS). In addition, patients who had negative radiographic progression tended to have better physical function than patients with zero or low positive progression scores. This finding gains importance in the light of the low radiographic progression rates that were found in the 2-year TEMPO trial.^14^ Despite these low progression rates, this (sensitive) longitudinal analysis suggests that any type of radiographic progression has repercussions for physical functioning. It may be argued that DAS and CRP inappropriately reflect disease activity and that the proposed association between radiographic progression and physical function would disappear if a variable that better reflects inflammatory activity were used. This is, however, highly unlikely. The contribution of radiographic progression to the HAQ score was similarly high before and after adjustment for DAS and CRP, so that both effects are truly separated (data not shown).

In a 10-year longitudinal study,^18^ we have previously observed that radiographic damage and disease activity independently contribute to changes in physical function in RA regardless of disease duration. Others have reported the longitudinal relationship between joint damage and physical function after adjustment for disease activity.^19^ In that particular study, performed on an inception cohort of patients with RA followed up for many years while receiving standard conventional antirheumatic treatment, the main driver of physical function was determined to be disease activity. But Welsing *et al* also found a significant contribution of joint damage (measured by van der Heijde’s modification of the Sharp score) to explain the variation in physical function.^19^

There are important differences between these previous studies^18^ ^19^ and our current study: First, we analysed a trial population using highly effective treatments. In our study, radiographic progression was minimal, although the level of baseline damage was, on average, substantial. Apart from that, the active trial treatment resulted in very low levels of disease activity in the majority of the trial population. Even with such a low level of variation in the determinants of function (disease activity and radiographic damage/progression) a relationship between radiographic damage progression and the HAQ score was suggested by this analysis. Second, in our study, we investigated the contribution of radiographic progression itself, rather than radiographic damage at any particular time point. Our analysis suggests that both the level of damage, and the progression rate, although very low, contributed to variation in physical function. Third, our observations were made in a 2-year follow-up study, whereas the previous studies^18^ ^19^ investigated function over a period of 9–12 years, in which deterioration of physical function is likely to be far more substantial. Taken together, however, the longitudinal studies provide evidence that physical function in patients with RA is determined not only by disease activity but also by radiographic damage and the rate at which radiographic damage increases over time. Fluctuations in the progression rate and in the disease activity cause demonstrable effects on physical function within very short time intervals. These data add to the ever-growing evidence that it is important not only to suppress disease activity but also to halt radiographic progression to maintain physical function.

It is not a coincidence that studies using longitudinal data analyses are consistent with the existence of a relationship between radiographic damage and physical function. It has been hypothesised that this relationship exists, but it has turned out to be rather difficult to confirm it statistically in studies using cross-sectional analytical approaches.^20^ ^21^ A longitudinal data analysis provides far more statistical power because it uses all available data of a prospectively followed cohort in an aggregated manner, while adjusting for spurious intra-patient correlation. Apart from that, longitudinal data analysis provides insight into how changes in disease activity and radiographic progression—for example, as a result of effective treatments, influence physical function.

Physical function is often considered to be a rather static outcome. However, recent experience from randomised clinical trials evaluating highly effective treatments has shown that impairment of physical function in patients with RA is reversible to some extent. In a recent analysis, Aletaha *et al* reported that the irreversible part of the HAQ score was between 0% and 33%, increasing with the duration of RA.^22^ We hypothesise that this irreversible impairment is caused by structural damage, while the reversible impairment is primarily inflammation. If true, early effective intervention to limit structural damage may be helpful in decreasing irreversible physical impairment.

Many studies support the use of early treatment of RA. Landewé *et al* showed that the best way to prevent radiographic damage is to start treatment before such damage has occurred.^23^ If this is not possible, aggressive treatment should be started within 12–24 months after the diagnosis of RA to limit radiographic progression over time.^23^ Anderson *et al* showed that the rate of radiological progression is set during the early stages of RA and suggested resetting this progression rate through pharmacological treatment as soon as possible after the disease is recognised.^24^ Other studies support the finding that radiographic damage occurs early in the onset of RA and progresses throughout the disease.^2^ ^3^ ^25^^–^27 A recent meta-analysis showed that starting treatment early has a long-term benefit on inhibition of structural damage.^28^ This analysis demonstrated that the association between radiographic progression and physical function was similarly important in relatively early RA (disease duration ⩽3 years) as compared with more advanced RA (disease duration >3 years), which emphasises the importance of minimising radiographic progression at all stages of the disease.

Rheumatologists frequently question whether the small differences in radiographic progression between trial arms are clinically meaningful. This analysis supports the view that subtle differences in radiographic progression have a measurable impact on physical function, although most probably at a level that is not truly clinically meaningful for the patient over a relatively short period. The question, however, is how physical function would be influenced if mild progression were allowed for years and years, using the argument that small fluctuations are not clinically meaningful.

## CONCLUSIONS

This analysis suggests that in patients with RA, greater radiographic damage and recent radiographic progression correlate with a higher degree of disability, after adjustment for age, sex, disease duration and disease activity.
